# Perceived COVID-19 risk is attenuated by ingroup trust: evidence from three empirical studies

**DOI:** 10.1186/s12889-021-10925-3

**Published:** 2021-05-05

**Authors:** Tegan Cruwys, Mark Stevens, Jessica L. Donaldson, Diana Cárdenas, Michael J. Platow, Katherine J. Reynolds, Polly Fong

**Affiliations:** Research School of Psychology, The Australian National University, Canberra, ACT 2601 Australia

**Keywords:** Social identity, Group processes, Trust, Health behavior, Risk perception

## Abstract

**Background:**

The social identity model of risk taking proposes that people take more risks with ingroup members because they trust them more. While this can be beneficial in some circumstances, in the context of the COVID-19 pandemic it has the potential to undermine an effective public health response if people underestimate the risk of contagion posed by ingroup members, or overestimate the risk of vaccines or treatments developed by outgroup members.

**Methods:**

Three studies (two prospective surveys, one experiment) with community-based adults tested the potential for the social identity model of risk taking to explain risk perception and risk taking in the context of COVID-19.

**Results:**

Study 1 was a two-wave study with a pre-COVID baseline, and found that people who identified more strongly as a member of their neighborhood pre-COVID tended to trust their neighbors more, and perceive interacting with them during COVID-19 lockdown to be less risky. Study 2 (*N* = 2033) replicated these findings in a two-wave nationally representative Australian sample. Study 3 (*N* = 216) was a pre-registered experiment which found that people indicated greater willingness to take a vaccine, and perceived it to be less risky, when it was developed by an ingroup compared to an outgroup source. We interpret this as evidence that the tendency to trust ingroup members more could be harnessed to enhance the COVID-19 response.

**Conclusions:**

Across all three studies, ingroup members were trusted more and were perceived to pose less health risk. These findings are discussed with a focus on how group processes can be more effectively incorporated into public health policy, both for the current pandemic and for future contagious disease threats.

## Background

In the face of the COVID-19 pandemic, governments have appealed to their populaces to engage in sustained behavior change *en masse* in an effort to reduce community infection rates. Effective and persuasive public health messaging has rarely (if ever) been more critical, and will remain so until vaccines have been effectively distributed worldwide.

A particular ongoing challenge has been persuading people to take precautions (e.g., physical distancing) within their closest social networks. Indeed, evidence points to a disconnect between the contexts that people *perceive* to be high risk and those where the majority of transmission is actually happening. Several epidemiological studies have found that the vast majority of COVID-19 transmission occurs in homes [1], particularly at informal gatherings of family or friends [2]. Despite this, people are most concerned that they will become infected outside their homes, viewing strangers as the greatest threat, and public transport and crowds as the most dangerous environments [3].

These risk perceptions have real consequences for the precautions people take. For example, one large Australian study that was conducted at the height of the country’s first COVID-19 wave in April 2020 found that while 84% of people were reducing their contact with strangers, only half were reducing contact in their workplace, and a mere 13% were reducing contact within their homes [4]. However, research to date has rarely distinguished between how people evaluate risk for different kinds of targets (e.g., friends, strangers) or in different contexts (e.g., work, home), instead focusing on global perceptions and protective behaviors [5, 6]. To enhance the success of public health messaging, we urgently require a framework that can explain the nuanced patterns of COVID-19 risk taking.

### A social identity approach to risk

Many of the behaviors recommended to reduce the spread of COVID-19 are fundamentally *social* behaviors (e.g., physical distancing; self-isolation). It follows then, that the science of social relationships is particularly relevant. One emergent social psychological framework that may help explain how people evaluate the risk of contagious disease is the *social identity model of risk taking* (SIMORT) [7, 8]*.* This model builds on the key principle of the social identity approach [9, 10]: that our sense of self-definition is derived not only from our individual traits and qualities, but also from the groups we categorize ourselves as members of—our social identities. Crucially too, our social identities are not mechanistically determined by our “objective” group memberships, but rather, we subjectively and dynamically define ourselves in ways that are psychologically meaningful in a particular social context [11]. A person might be a resident of a neighborhood but feel no subjective affinity with it. Or, in contrast, a person might have a strong sense of identification with a neighborhood despite no longer residing there.

When a person does define themselves in terms of a particular social identity, this has cognitive, emotional, and behavioral consequences. Indeed, our perception of the world (including, but not limited to, our perception of health risk) is inextricably linked with how we define ourselves in relation to our own and other social groups (e.g., as a man, as a Canadian, or as a farmer) [12, 13]. Furthermore, when a particular social identity becomes salient to us, this affects how we perceive other people – as fellow ingroup members, or as outgroup members. A large body of work has demonstrated that ingroup members are extended a variety of benefits, such as more positive interpersonal evaluations and greater trust, cooperation, and support [14–17]. This is particularly true for members of groups that are most important to us (i.e., those groups with which we strongly identify).

Of these various benefits that tend to be extended to ingroup members over outgroup members, one of them – greater trust – might have something of a dark side. Indeed, SIMORT argues that, because ingroup members are typically trusted to a greater degree than outgroup members, this has systematic effects on the degree to which we see ingroup members (versus outgroup members) as a potential threat. Whether in the context of financial risk (e.g., “affinity fraud”) [18], corporate espionage, or most relevant for our purposes here, contagious disease, people tend to use shared group membership as a heuristic for ‘safety’ and are thus more likely to behave in ways that place their fate ‘in the hands’ of ingroup members. It follows from SIMORT, then, that social identity processes are central to an effective COVID-19 response because people systematically overestimate risks associated with interacting with outgroup members, while underestimating risks associated with interacting with ingroup members.

Initial evidence for SIMORT comes from a variety of domains spanning large scale field studies at mass gatherings, longitudinal evaluations at festivals, through to controlled experimental laboratory studies. For example, American university students perceived drinking beer from cans emblazoned with their university logo to be less dangerous than from cans where such labelling was absent [19]. Along similar lines, in a sample of over 1300 young people at an Australian school leavers festival, researchers found that those who strongly identified with other festival attendees reported greater trust in fellow attendees and, in turn, rated as less risky behaviors such as sharing drinks, unprotected sex, or walking home alone while at the festival [7]. Another field study conducted at music festivals in the United Kingdom found that those who identified with fellow attendees reported reduced vulnerability to disease and a greater likelihood of engaging in health risk behaviors [20].

Finally, there is experimental evidence for SIMORT. One experimental study found that the need for personal space is attenuated when one shares a social identity with others, such that English university students in two lab experiments placed chairs closer together when their anticipated discussion partner was an ingroup member rather than an outgroup member [21]. Another experiment randomly assigned Australian university students to either a “green” or “red” group (ostensibly on the basis of their color perception). Participants were asked to complete a Lego model that they were told had been commenced by a previous participant from the green group (i.e., either an ingroup or outgroup member). The experiment also manipulated disease salience, with half of the participants encountering what appeared to be used tissues in the workspace, and the experimenter stating that the previous participant had a cold. After completing the Lego task, participants reported that they had taken a greater risk with their health in the disease salient conditions, but *only* when the previous participant was an outgroup member [7]. This suggests that in a context where the threat of contagious disease is salient, people are less sensitive to this risk when the source is an ingroup member.

### The current research

Although this evidence is suggestive, the applicability of SIMORT to the COVID-19 pandemic is not yet clear. The context in which people evaluate the risk of social interaction has fundamentally changed, and pre-COVID research is unlikely to have fully captured the determinants of relatively novel health behaviors such as physical distancing. Therefore, the current program of research sought to urgently fill this gap and contribute to the COVID-19 response. The primary hypothesis, assessed across all three studies with Australian participants, was that shared group membership will increase trust in members of the ingroup, which, in turn, will reduce COVID-19 related risk perception and increase COVID-19 related risk taking.

## Study 1

Study 1 sought to examine this hypothesis in a community sample of people who expressed interest in getting involved in a social participation campaign in their local neighborhood. This two-wave survey was collected in collaboration with community not-for-profit organization Relationships Australia, with the original goal of providing an evaluation of *Neighbour Day* – a grassroots community participation initiative to increase social participation and social cohesion. However, given that (a) the baseline survey was distributed in the 2–4 weeks before the COVID-19 pandemic reached Australia, and (b) Neighbor Day could not take place in its original form due to severe local restrictions, this project was redesigned prior to the Time 2 follow-up to enable the investigation of the present research question.

### Method

Participants were 97 community members (78 female, 18 male, 1 did not disclose) originally recruited prior to the pandemic via the Relationships Australia website (https://neighbourday.org/) and a mailing list of people who had expressed interest in the *Neighbour Day* campaign. Participants were aged 23 to 76 (*M* = 46.33; *SD* = 14.79) and were located in the seven different states and territories of Australia, with 79 different postcodes represented. Participants at Time 1 (T1) were entered into a prize draw to win one of two AUD$200 gift vouchers. At Time 2 (T2), all participants received a AUD$30 gift voucher.

T1 was completed in early March 2020 (81% in the first 3 days of March), prior to community spread of COVID-19 in Australia or the implementation of any local restrictions. T2 was collected from 6 to 30 April, during the most extreme national COVID-19 restrictions, including a stay-at-home order, forced closure of dine-in restaurants and cafes, and strict limits on household visits. However, daily exercise was permitted, including with people from other households if physical distancing was maintained.

#### Measures

##### Neighborhood social identification

At T1, the four item social identification scale was included (FISI) [22], with items such as “I see myself as a resident of this neighborhood” measured on a seven point scale from strongly disagree [1] to strongly agree [7], α = .84.

##### Trust

A one-item measure of ingroup trust adapted from prior research [23] was included: “People in this neighborhood can be trusted”, which was rated on a seven-point scale from strongly disagree [1] to strongly agree [7].

##### Perceived risk of interaction with neighbors during lockdown

A single item was adapted from previous risk research [7] to evaluate the perceived risk of interacting with neighbors during lockdown: “How safe or unsafe do you feel while interacting with people in your neighborhood?”, which was measured on a seven-point scale from very unsafe [1] to very safe [7]. To aid interpretation, this item was reversed such that high scores represented a greater degree of perceived risk.

### Results

**Fig. 1:**
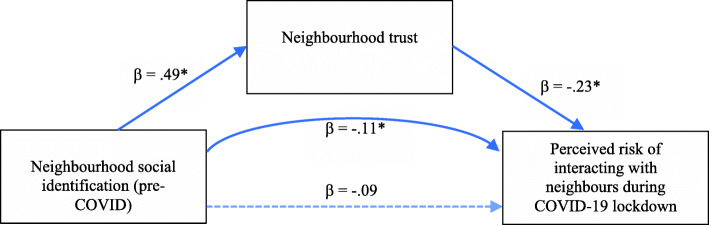
Neighborhood identification indirectly predicted reduced perception that neighbors were a risk during COVID-19 lockdown via trust. Note. The curved line arrow in the figure represents the hypothesized indirect effect. The total effect of social identification on risk perception was β = −.20, *p* = .052

To evaluate the hypothesis, a mediation model was specified in PROCESS (v.3.3) [24] with 5000 bootstrapped samples in which the predictor variable was T1 neighborhood identification, the mediator was T2 neighborhood trust, and the dependent variable was T2 perceived risk of neighbor interaction. The key test of the hypothesis was a significant indirect effect, indicated by a 95% confidence interval that did not cross zero [24]. T1 neighborhood identification significantly and positively predicted T2 neighborhood trust, β = .49, *p* < .001. T2 neighborhood trust was, in turn, associated with lower T2 perceived risk of neighbor interaction, β = −.23, *p* = .045. Supporting the hypothesis, the indirect effect of neighborhood identification on perceived risk of neighbor interaction via trust was significant, β = −.11 (95% CI: −.23, −.005). The model is summarized in Fig. 1.

### Discussion

Study 1 found that people who identified more strongly as a member of their neighborhood prior to the COVID-19 pandemic were more likely to trust their neighbors during lockdown, and thus, perceived interacting with their neighbors during lockdown to be safer. A major strength of Study 1 was its pre-COVID baseline, which sets it apart from the majority of COVID-19 research and increases our confidence that our measure of neighborhood identification was ‘uncontaminated’ by the swift social and economic changes that occurred in response to the pandemic. However, limitations were its relatively small sample size and the fact that a baseline measure of risk was not available. Study 2 sought to address these limitations.

## Study 2

Study 2 was a multi-wave nationally representative survey of social psychological determinants of COVID-19 related behaviors. Study 2 sought to replicate Study 1 with several improvements including a large sample size, baseline measures of all variables, and a more behaviorally-oriented dependent variable. Furthermore, risk aversion toward other groups (not just the focal ingroup) was also measured, to assess whether the effect of social identity processes on risk were (as hypothesized) specific to the ingroup. This is important because it is also possible that social identification is associated with generalized risk perception, whereas SIMORT proposes a specific effect on the risk arising from ingroup (vs. outgroup) members. Study 2 occurred entirely during the period of COVID-19 restrictions, and so it was possible to analyze the data using a completely lagged design in which social identification with the neighborhood predicted *change* in trust and, indirectly, *change* in perceived COVID-19 risk.

### Method

Participants (*N* = 2033) were Australian residents stratified by age, sex, ancestry, and income. Recruitment was conducted using a Qualtrics panel, with participants paid the standard panel rate for their time. The sample was aged from 18 to 87 (*M* = 49.90; *SD* = 16.80), and comprised 1071 women (56.6%), 960 men (47.2%), and 4 other (0.2%). Due to the sampling strategy, the sample was diverse in geographic location (with all regions of Australia represented proportional to population), educational attainment, income, and ancestry.

T1 was collected between 11 May and 27 May 2020, at a time when Australian governments (both federal and state-level) were beginning to reduce some of the strict limitations that had been established throughout March (e.g., restaurants were reopening with strict physical distancing limits). T2 (collected between 16 June and 16 July) was completed 2 weeks after restrictions were further eased (e.g., gatherings of up to 20 people were allowed) to examine whether people maintained physical distancing when these behaviors were perhaps most important – as formal restrictions were reduced and the risk of community transmission was increasing.

#### Measures

##### Neighborhood social identification

The single item social identification scale (SISI; 22) was used to measure neighborhood social identification, as follows: “I identify as a member of my neighborhood” on a seven-point scale from strongly disagree [1] to strongly agree [7]. This scale has been validated and widely used, with evidence suggesting it has comparable psychometric properties to longer social identification scales [22, 25].

##### Trust

A single item was used: “In general, I trust my neighbors” with response options from do not trust at all [1] to trust unconditionally [7]. This one-item trust measure was adapted from a validated single-item general trust scale, which has been widely used in population surveys [26].

##### Physical distancing from neighbors during lockdown

Study 2 used a more behaviorally-oriented indicator of risk taking (vs. risk aversion): the degree of actual physical distancing from neighbors that participants indicated that they engaged in. Participants were given the following background: “Social distance rules endorsed by the Australian government stipulate a distance of 1.5 meters between yourself and others. But really it is you that decides how much distance you keep from different people. Imagine walking down the street, how much distance do you think you would keep between yourself and the other persons listed below?” Different options were then presented including “a neighbor” (our focal group for this analysis), as well as “a person from another neighborhood”, “a stranger” and “a close friend”. Response options ranged from no distance [1] to more than 2.5 m [7]. Visual indicators of the distance between two people, modelled on local public health messaging, were included to aid interpretability.

### Results

To evaluate the hypothesis, a mediation model was specified in PROCESS (v.3.3) [24] with 5000 bootstrapped samples in which the predictor variable was T1 neighborhood identification, the mediator was T2 neighborhood trust, and the dependent variable was T2 physical distancing from neighbors. In addition, the covariates of T1 neighborhood trust and T1 physical distancing were also included, such that residuals (change over time) in the mediator and dependent variable were the focus of the analysis.

**Fig. 2:**
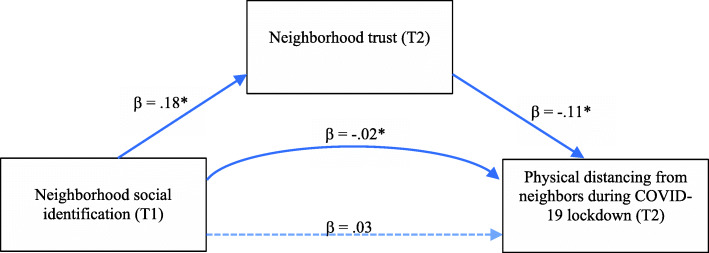
Neighborhood identification indirectly predicts reduced physical distancing with neighbors during COVID-19 lockdown via trust. This model was adjusted for baseline covariates of T1 neighborhood trust and T1 physical distancing from neighbors. Therefore, this model illustrates that baseline neighborhood social identification predicts change in trust and physical distancing over time. Note. The curved line arrow in the figure represents the hypothesized indirect effect. The total effect of social identification on risk perception was β = .00, *p* = .757

T1 neighborhood identification significantly and positively predicted T2 neighborhood trust, β = .18, *p* < .001, and T2 neighborhood trust was, in turn, significantly associated with reduced T2 physical distancing, β = −.11, *p* < .001. Supporting the hypothesis, the indirect effect of neighborhood identification on physical distancing via trust was significant, β = −.02 (95% CI: −.03, −.01). The model is summarized in Fig. 2.

#### Sensitivity analysis

As an additional test of the model, we calculated the dependent variable as the *difference* between the degree of physical distancing from (a) a person in one’s own neighborhood and (b) a person from another neighborhood. If significant, this analysis would suggest that the social identification is not related to a general tendency to physically distance from everyone (or not), but rather its effects on risk are, as hypothesized, *specific* to the social group in question. This would strengthen our confidence that this is a social identity phenomenon.

In this analysis, which in line with the primary model included T1 covariates, the effect of T1 neighborhood identification predicted T2 neighborhood trust (β = .18, *p* < .001), and trust predicted the difference between distancing towards neighbors versus non-neighbors at T2 (β = −.08, *p* = .008). The indirect effect was significant, β = −.01 (95% CI: −.026, −.003).

A final follow up analysis sought to evaluate whether the effects were specific to neighbors or generalizable to other groups by replacing the dependent variable and associated covariates with (a) people from other neighborhoods, (b) a stranger, and (c) a close friend. In no case was there a significant indirect effect, or an effect of T2 neighborhood trust on physical distancing.

### Discussion

Study 2 replicated the results of Study 1 and built on them in several ways. First, it was able to control for baseline levels of neighborhood trust and physical distancing, which increased our confidence in the direction of the hypothesized effects. Second, in Study 2 the measure of COVID-19 risk was based on self-reported behavior of a key COVID-19 preventative measure: degree of physical distancing. This increases our confidence that these psychological processes have public health implications. The hypothesized indirect effect and focal model pathways, although significant, were substantially smaller in Study 2 than in Study 1. This is likely due, at least in part, to the inclusion of covariates (i.e., because we modelled the relationships between residuals). However, another possibility is that the effects are more modest in size for risk taking compared to risk perception. A final strength of Study 2 was the inclusion of measures of physical distancing with non-focal (out) groups, and we found that the effect of ingroup trust was specific to fellow ingroup members, and did not extend to other groups (including people from other neighbors, strangers, or close friends).

## Study 3

Study 3 sought to extend our analysis in two ways. First, it utilized an experimental design to provide the strongest test of the causal role of ingroup trust on COVID-19 risk. Second, it utilized validated multi-item measures of trust and risk behavior. Third, it included a control condition, with a view to investigating the inferences people make about trust and risk in the absence of explicit information about group membership. Fourth and finally, Study 3 aimed to provide an initial test of how the link between ingroup trust and COVID risk might be fruitfully used to inform the public health response. We reasoned that one of the greatest challenges of behavior change still ahead in combating the pandemic is persuading the global population to accept a COVID-19 vaccine. All vaccines have some (usually extremely small) risk associated with them, and these potential risks are often amplified and given undue emphasis by vaccine sceptics [27, 28]. In line with our theoretical model, we reasoned that people will perceive a vaccine as less risky, and indicate a greater willingness to take the vaccine, if it has been developed and endorsed by ingroup (rather than outgroup) members because of the greater trust we have for them.

### Method

Participants were 216 Australian residents recruited for an online experiment via *Prolific*, an academic research web platform, between 13 and 17 August 2020 – when hundreds of vaccines were in development, but results of phase 3 trials were not yet available. Participants were 108 men and 104 women, with four people indicating that they were non-binary or choosing not to disclose their gender. Participants ranged in age from 18 to 72 (*M* = 30.67; *SD* = 10.55) and were diverse in terms of their educational attainment. Participants were paid the standard Prolific rate for their time (approximately AUD$8.30/h).

Study 3 was an experiment with three conditions: ingroup source, outgroup source, and control. Participants were asked to consider a hypothetical scenario in which a COVID-19 vaccine was announced in a press conference by Australia’s medical officials. Participants first completed a social identification scale to make their social identity as an Australian salient.

Participants were then asked to imagine that the following scenario in a speech attributed to Australia’s chief medical officer. The control condition excluded the text in square brackets and did not attribute the vaccine or the risk evaluation to any particular group.

*“I am pleased to announce that a first vaccine for COVID-19 will, from tomorrow, be available to the Australian public.*

*The vaccine was developed by [Australian/French] scientists and has passed the final stage of testing. It showed an 85% success rate in preventing the virus and carries only a 2% risk of serious side effects, which [our/France’s] leading health experts consider an acceptable level of risk.*

*Of course, this vaccine is being made available around the world, including in Australia. A website has just gone online where everyone can sign up to receive the vaccination, and indicate the doctor’s surgery where they would like to receive it.*

*This will be the first stage in rolling out the vaccine to all Australians.”*

French people were selected as the outgroup for several reasons. We sought a real-world outgroup which was perceived by Australians to be of similar status (e.g., unlike the United States which is typically perceived as higher status) and to be a nation about which Australians tend to have relatively neutral stereotypes (e.g., unlike Canada, which is typically perceived very positively).

The design, measures, hypotheses and analyses were pre-registered (https://aspredicted.org/h8qj9.pdf). The pre-registration also included a power analysis, which indicated a recruitment target of 250 to achieve a minimum sample size of 207 for analyses.

#### Measures

##### Trust

In Study 3, trust was measured using a comprehensive validated scale adapted from previous research [29]. As well as some items related to general trust, this measure included subscales relating to perceived integrity (e.g., “Sound principles seem to guide [Australian/French] scientists’ behavior”), benevolence (e.g., “[Australian/French] scientists would not knowingly do anything to hurt me”) and competence (e.g., “I feel very confident about [Australian/French] scientists’ skills”), each rated on a scale from disagree strongly [1] to agree strongly [5]. The underlined content was removed in the control condition. Two of the items from the original scale could not be readily adapted to the experimental context of Study 3, yielding a 19-item scale [7]. The scale has primarily been used as a single unitary indicator of trust in prior research [30] and the overall reliability (α = .91) suggested this was also appropriate here.

##### Perceived COVID-19 vaccine risk

Perceived risk of the proposed vaccine and willingness to take the vaccine was measured using four items adapted from prior research [7], each measured on a continuous sliding scale from not at all [0] to extremely [100]. The items were “How risky do you think receiving the vaccine is?”, “How safe do you think having the vaccine is?”, “How risky do you think not having the vaccine is?”, and “How likely is it that you will sign up for the vaccine?”. Three items were reversed such that higher scores represented a greater perceived risk (and avoidance) of the hypothetical vaccine (α = .86). In addition to the Cronbach’s alpha, the unitary structure of these items was further supported by an exploratory factor analysis, which revealed only one factor with an eigenvalue > 1 that accounted for 70% the variance, with each item loading onto this factor at >.72.

##### Manipulation check

At the end of the study, participants were asked “In the scenario you were asked to imagine, which scientists had developed the vaccine?” with response options of “Australian”, “French”, “American”, “I can’t remember” and “It didn’t say which scientists developed the vaccine”. Participants in the ingroup and outgroup conditions were required to accurately identify the nationality of the scientists in their vignette to pass the manipulation check, while either of the final two options were acceptable to pass the manipulation check in the control condition.

### Results

Initially, 250 people completed the study, however, in accordance with our pre-registered data management plan, 34 who failed the manipulation check were excluded. This left 216 participants in the final sample.

**Fig. 3:**
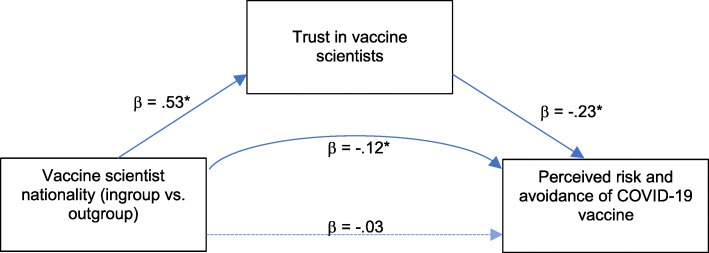
Shared group membership with vaccine scientists indirectly predicts reduced perceptions of vaccine risk and increased willingness to take the vaccine via trust. Note. The curved line arrow in the figure represents the hypothesized indirect effect. The total effect of shared group membership on risk was β = −.09, *p* = .565

The hypothesis was tested using PROCESS [24] with 5000 bootstrapped samples. Experimental condition was entered as the (categorical) independent variable, trust in scientists was the mediator, and perceived vaccine risk was the dependent variable. All combinations of contrast coding were used in the mediation analysis such that comparisons were examined between each pair of conditions. Of these, only those comparing the ingroup source to the outgroup source were significant. This model is presented in Fig. 3.

Participants in the ingroup condition expressed greater trust in the vaccine scientists, β = .53, *p* < .001. Trust in vaccine scientists, in turn, predicted a reduction in perceived risk and avoidance of the COVID-19 vaccine, β = −.23, *p* = .005. Supporting the hypothesis, the indirect effect was significant, β = −12. (95% CI: −.27, −.02).

**Fig. 4:**
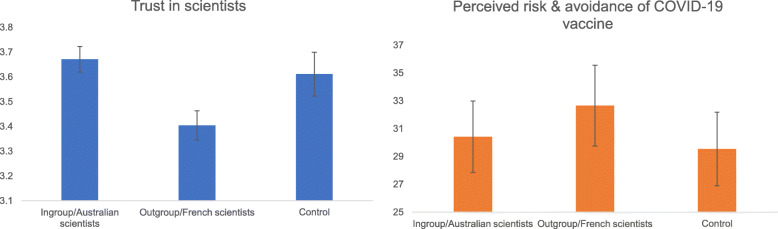
Estimated marginal means for each of the three Study 3 conditions on trust in vaccine scientists (the hypothesized mediator) and perceived risk and avoidance of COVID-19 vaccine (the hypothesized outcome). Error bars represent standard error

Comparisons between the control condition and each of the group membership conditions revealed no significant effects except the effect of trust on risk, which was robust in all conditions. However, inspection of the estimated marginal means (presented in Fig. 4) indicated that, rather than falling between the ingroup and outgroup conditions, the control condition most closely resembled the ingroup condition, albeit with a slightly wider variance that rendered its comparison with the outgroup condition nonsignificant.

### Discussion

Study 3 provided several new insights. It replicated the previous studies in finding that ingroup trust is a key predictor of COVID-19 risk. However, Study 3 focused on a different domain of risk: perceived riskiness of and willingness to receive a COVID-19 vaccine. This is important because it suggests ways in which the general tendency towards ingroup trust might be utilized for effective public health messaging – by emphasizing that vaccine development is an *ingroup initiative* being spearheaded by ingroup members. Of course, for many people around the world, the scientists developing COVID-19 vaccines will be outgroup members (based on nationality). However, shared identity can nevertheless be emphasized on dimensions other than nationality, potentially encompassing all of humanity [31, 32].

Study 3 had several strengths over the prior studies, including its pre-registered experimental design and its use of more comprehensive validated measures of the constructs of interest. However, a limitation of Study 3 was that the findings may have been influenced by the specific ingroup and outgroup selected (Australia and France), and the real-world intergroup dynamics of these groups driven by normative content and status. The effects may have been quite different if, for example, a high status country (e.g., the United States) had been selected as the outgroup. Findings should thus be generalized with caution, and with consideration of these dynamics. This limitation is best addressed in research by employing a minimal group paradigm, where the groups in question have no real-world “baggage” associated with them. Although this was not possible in the context of the present study, previous research has shown that the effects proposed in SIMORT also emerge in the context of such paradigms [7]. The comparison to the control condition provided some tentative evidence that in the absence of group membership information, participants may infer a shared group membership – at least in the context of a local vaccine roll-out or similar public health initiative. This is promising for intervention efforts, because it suggests that in countries where vaccine developers are an outgroup, de-emphasizing the nation of origin may reduce vaccine hesitancy.

## General discussion

Across three studies, we have found evidence that COVID-19 risk and preventative behaviors are fundamentally structured by our group memberships – and most pertinently, by the greater trust that we afford fellow ingroup members. Study 1 demonstrated that people who strongly identified as a member of their neighborhood prior to the pandemic trusted their neighbors more during lockdown, and felt that interacting with them would be safer. Study 2 replicated this in a nationally representative sample of over 2000 people, finding that neighborhood social identification predicted positive change in neighborhood trust, which in turn predicted reduced physical distancing from neighbors. Study 2 also demonstrated that this phenomenon was sensitive to the specific group membership of the target group, with physical distancing from strangers, close friends, or people from other neighborhoods unaffected by neighborhood trust. Study 3 was a pre-registered experiment that sought to test whether it might be possible to harness these social identity processes in a positive way: to reduce perceived risk of a COVID-19 vaccine and increase willingness to receive it. Here, we found that participants who considered a vaccine developed by ingroup scientists (compared to outgroup scientists) were more likely to trust those scientists and, in turn, were less likely to perceive the vaccine as risky and were more willing to receive it.

### Theoretical implications

The evidence presented here speaks to the nuanced understanding of group processes, and trust in particular, that is needed to accurately model health behavior. Of course, ingroup trust has many benefits, such as facilitating cooperation, helping, and, particularly relevant in this context, solidarity in the COVID-19 response [3]. However, ingroup trust also has some very concrete downsides, as demonstrated here. Similarly, there may well be situations in which the tendency to overestimate risk of outgroup members and underestimate risk of ingroup members is also beneficial. Indeed, this is a key argument of behavioral immune system researchers, who argue that people are largely accurate in their heuristic that outgroup members are a more likely source of disease [33, 34]. Nevertheless, these studies illustrate that knowledge of group processes is crucial to the capacity of researchers, health professionals, and policymakers to accurately predict – and effectively influence – COVID-19 related risk behaviors.

The findings also speak to the utility of behavioral science, and social psychology in particular, to provide meaningful recommendations for tackling real-world problems. Indeed, as others have argued, this may be most especially true in times of crisis [35–37]. Although the COVID-19 pandemic precipitated an unprecedented investment in vaccine and treatment research (which is, of course, crucially important), investment in behavioral science has been more muted. We argue this represents a lost opportunity at a time where behavioral science is proving crucial to effective decision making and communication [38].

### Practical implications

There are several implications of these findings for the practical management of the COVID-19 pandemic (and indeed, future contagious disease threats). First, they suggest that a particularly beneficial focus for public health messaging may be emphasizing the dangers posed by the people we are closest too (e.g., friends, family, and those in one’s neighborhood). People require very little encouragement to avoid strangers and outgroup members. However, the perceived risk is lower in one’s closest networks, and even if people are aware of these risks, they may be more likely to consider them worth taking. Therefore, investment is needed in messaging to provide people with guidance about how gatherings with loved ones can be made safer.

Similarly, uptake of COVID-19 vaccination (or indeed, other novel health behaviors) is likely to be greatest when promoted or developed by ingroup members – who are perceived to be more trustworthy. “We are all in this together” has become a cliché in the COVID-19 pandemic, but it is a wise and effective government that is able to not only *claim* this axiom, but also *embody* it consistently in its public health messaging [39].

### Limitations and future directions

One notable feature of these studies is that they were all conducted in Australia, a country that has to date been relatively successful, globally speaking, in its COVID-19 response. COVID-19 risk in the community is therefore objectively lower than many countries, and risk perception may also be attenuated. However, it is worth noting that all three studies included participants based in Melbourne, which had recorded over 20,000 cases and 820 deaths at the time of writing and endured one of the longest and most restrictive lockdowns in the world. Therefore, it is not the case that Australia has been entirely spared by the pandemic. Future studies examining these effects might be particularly compelling if they were able to demonstrate that rates of COVID-19 transmission were affected by one’s subjectively assessed group membership with, and trust in, the index case. Importantly, though, we would not expect overall differences in likelihood of transmission to differ between “low” and “high” identifiers with any particular social group. Rather, social identity can explain why *certain* people take risks with *certain* targets – and it is this more nuanced perspective on risk that warrants continued investigation in future research.

## Conclusions

COVID-19 has become a pandemic when many other candidate viruses did not. This, we contend, is at least partly because it exploited fundamental features of human psychology: our strong desire for human contact, and our willingness to discount risk associated with our closest companions. In three studies, this paper found that our willingness to take risks associated with COVID-19 transmission (e.g., be in close proximity with others; be vaccinated) is greater when the source of these risks is perceived to be a member of one’s own valued social group. This tendency was fully mediated by our tendency to trust ingroup members, even in circumstances where this might be detrimental to our health. To tackle this and future pandemics more effectively, it is vital that public health messaging effectively communicates that transmission risk is often greatest with the people we care most about.

## Data Availability

The datasets used and/or analysed during these studies will be made available in the OSF repository upon publication, https://osf.io/upvnw/

## Abbreviations

CI: Confidence interval
COVID-19: Coronavirus disease of 2019
FISI: Four item social identification
M: Mean
SIMORT: Social identity model of risk taking
SD: Standard deviation
SISI: Single item social identification
T1: Time 1
T2: Time 2

## References

[1] Madewell ZJ, Yang Y, Longini IMJ, Halloran E, Dean NE. Household transmission of SARS-CoV-2: a systematic review and meta-analysis of secondary attack rate. medRxiv Prepr. 2020. 10.1101/2020.07.29.20164590.

[2] Qian G, Yang N, Ma AHY, Wang L, Li G, Chen X, Chen X (2020). A COVID-19 transmission within a family cluster by presymptomatic infectors in China. Clin Infect Dis.

[3] Jetten J, Reicher SD, Haslam SA, Cruwys T (2020). Together apart: the psychology of COVID-19.

[4] Liddy M, Hanrahan C, Byrd J (2020). How Australians feel about the coronavirus crisis and Scott Morrison’s response.

[5] Olivera-La Rosa A, Chuquichambi EG, Ingram GPD (2020). Keep your (social) distance: pathogen concerns and social perception in the time of COVID-19. Pers Individ Dif.

[6] Maaravi Y, Heller B (2020). Not all worries were created equal: the case of COVID-19 anxiety. Public Health.

[7] Cruwys T, Greenaway KH, Ferris LJ, Rathbone JA, Saeri AK, Williams E, Parker SL, Chang MXL, Croft N, Bingley W, Grace L (2020). When trust Goes wrong: a social identity model of risk taking. J Pers Soc Psychol.

[8] Cruwys T, Stevens M, Greenaway KH (2020). A social identity perspective on COVID-19: health risk is affected by shared group membership. Br J Soc Psychol..

[9] Tajfel H, Billig MG, Bundy RP, Flament C (1971). Social categorization and intergroup behaviour. Eur J Soc Psychol.

[10] Turner JC, Hogg MA, Oakes PJ, Reicher SD, Wetherell MS (1987). Rediscovering the social group: a self-categorization theory.

[11] Onorato RS, Turner JC (2004). Fluidity in the self-concept: the shift from personal to social identity. Eur J Soc Psychol.

[12] Abrams D, Wetherell MS, Cochrane S, Hogg M a, Turner JC (1990). Knowing what to think by knowing who you are: self-categorization and the nature of norm formation, conformity and group polarization. Br J Soc Psychol.

[13] Jetten J, Haslam SA, Cruwys T, Greenaway KH, Haslam C, Steffens NK (2017). Advancing the social identity approach to health and well-being: progressing the social cure research agenda. Eur J Soc Psychol.

[14] Greenaway KH, Wright RG, Willingham J, Reynolds KJ, Haslam S (2015). Shared identity is key to effective communication. Personal Soc Psychol Bull.

[15] Tanis M, Postmes T (2005). A social identity approach to trust: interpersonal perception, group membership and trusting behaviour. Eur J Soc Psychol.

[16] Brewer MB, Krueger JI (2008). Depersonalized trust and ingroup cooperation. Rationality and social responsibility: essays in Honor of Robyn Mason Dawes. Modern pioneers in psychological science: an APS-psychology press series.

[17] Platow MJ, Foddy M, Yamagishi T, Lim LI, Chow A (2012). Two experimental tests of trust in in-group strangers: the moderating role of common knowledge of group membership. Eur J Soc Psychol.

[18] Blois K, Ryan A (2013). Affinity fraud and trust within financial markets. J Financ Crime.

[19] Loersch C, Bartholow BD (2011). The color of safety: Ingroup associated colors make beer safer. J Exp Soc Psychol.

[20] Hult Khazaie D, Khan SS (2019). Shared social identification in mass gatherings lowers health risk perceptions via lowered disgust. Br J Soc Psychol..

[21] Novelli D, Drury J, Reicher S (2010). Come together: Two studies concerning the impact of group relations on “personal space”. Br J Soc Psychol..

[22] Postmes T, Haslam SA, Jans L (2013). A single-item measure of social identification: reliability, validity, and utility. Br J Soc Psychol..

[23] Industry CM of. *General Social Survey (Catalogue No. 89F0115X).*; 2013. https://www150.statcan.gc.ca/n1/en/pub/89f0115x/89f0115x2013001-eng.pdf?st=GlLmgrc7.

[24] Hayes AF (2017). Introduction to mediation, moderation, and conditional Process analysis: a regression-based approach.

[25] Reysen S, Katzarska-miller I, Nesbit SM, Pierce L (2013). Further validation of a single-item measure of social identification. Eur J Soc Psychol.

[26] Helliwell JF, Wang S. Trust and Wellbeing. Cambridge; 2010. http://www.nber.org/papers/w15911

[27] Nihlén FJ (2018). Vaccine hesitancy and trust. Ethical aspects of risk communication. Scand J Public Health.

[28] Browne M (2018). Epistemic divides and ontological confusions: the psychology of vaccine scepticism. Hum Vaccines Immunother.

[29] Mayer RC, Davis JH (1999). The effect of the performance appraisal system on trust for management: a field quasi-experiment. J Appl Psychol.

[30] Schoorman FD, Mayer RC, Davis JH (2007). An integrative model of organiszational trust: past, present, and future. Acad Manag Rev.

[31] Greenaway KH, Louis WR (2010). Only human: hostile human norms can reduce legitimization of intergroup discrimination by perpetrators of historical atrocities. Br J Soc Psychol..

[32] Wohl MJA, Branscombe NR (2005). Forgiveness and collective guilt assignment to historical perpetrator groups depend on level of social category inclusiveness. J Pers Soc Psychol.

[33] Murray DR, Schaller M (2016). The behavioral immune system: implications for social cognition, social interaction, and social influence. Adv Exp Soc Psychol.

[34] Schaller M, Park JH (2011). The behavioral immune system (and why it matters). Curr Dir Psychol Sci.

[35] Elcheroth G, Drury J (2020). Collective resilience in times of crisis: lessons from the literature for socially effective responses to the pandemic. Br J Soc Psychol..

[36] Bavel JJV, Baicker K, Boggio PS, Capraro V, Cichocka A, Cikara M, Crockett MJ, Crum AJ, Douglas KM, Druckman JN, Drury J, Dube O, Ellemers N, Finkel EJ, Fowler JH, Gelfand M, Han S, Haslam SA, Jetten J, Kitayama S, Mobbs D, Napper LE, Packer DJ, Pennycook G, Peters E, Petty RE, Rand DG, Reicher SD, Schnall S, Shariff A, Skitka LJ, Smith SS, Sunstein CR, Tabri N, Tucker JA, Linden S, Lange P, Weeden KA, Wohl MJA, Zaki J, Zion SR, Willer R (2020). Using social and behavioural science to support COVID-19 pandemic response. Nat Hum Behav.

[37] Bonell C, Michie S, Reicher S, West R, Bear L, Yardley L, Curtis V, Amlôt R, Rubin GJ (2020). Harnessing behavioural science in public health campaigns to maintain “social distancing” in response to the COVID-19 pandemic: key principles. J Epidemiol Community Health.

[38] Freedman L (2020). Scientific advice at a time of emergency. SAGE and Covid-19. Polit Q.

[39] McGuire D, Cunningham JEA, Reynolds K, Matthews-Smith G (2020). Beating the virus: an examination of the crisis communication approach taken by New Zealand prime minister Jacinda Ardern during the Covid-19 pandemic. Hum Resour Dev Int.

